# Hair mercury levels in Amazonian populations: spatial distribution and trends

**DOI:** 10.1186/1476-072X-8-71

**Published:** 2009-12-21

**Authors:** Flavia L Barbieri, Jacques Gardon

**Affiliations:** 1 IRD - SELADIS, La Paz, Bolivia; 2 IRD - HSM, La Paz, Bolivia

## Abstract

**Background:**

Mercury is present in the Amazonian aquatic environments from both natural and anthropogenic sources. As a consequence, many riverside populations are exposed to methylmercury, a highly toxic organic form of mercury, because of their intense fish consumption. Many studies have analysed this exposure from different approaches since the early nineties. This review aims to systematize the information in spatial distribution, comparing hair mercury levels by studied population and Amazonian river basin, looking for exposure trends.

**Methods:**

The reviewed papers were selected from scientific databases and online libraries. We included studies with a direct measure of hair mercury concentrations in a sample size larger than 10 people, without considering the objectives, approach of the study or mercury speciation. The results are presented in tables and maps by river basin, displaying hair mercury levels and specifying the studied population and health impact, if any.

**Results:**

The majority of the studies have been carried out in communities from the central Amazonian regions, particularly on the Tapajós River basin. The results seem quite variable; hair mercury means range from 1.1 to 34.2 μg/g. Most studies did not show any significant difference in hair mercury levels by gender or age. Overall, authors emphasized fish consumption frequency as the main risk factor of exposure. The most studied adverse health effect is by far the neurological performance, especially motricity. However, it is not possible to conclude on the relation between hair mercury levels and health impact in the Amazonian situation because of the relatively small number of studies.

**Conclusions:**

Hair mercury levels in the Amazonian regions seem to be very heterogenic, depending on several factors. There is no obvious spatial trend and there are many areas that have never been studied. Taking into account the low mercury levels currently handled as acceptable, the majority of the Amazonian populations can be considered exposed to methylmercury contamination. The situation for many of these traditional communities is very complex because of their high dependence on fish nutrients. It remains difficult to conclude on the Public Health implication of mercury exposure in this context.

## Background

Since the Minamata tragedy as well as the Basra incident, methylmercury contamination has been a source of concern worldwide. There has been extensive work attempting to assess the human health impact of mercury contamination through fish, seafood or marine mammal consumption, such as the Seychelles and Faroe Islands birth cohorts and the New Zealand study [1-8].

In the Amazonian aquatic environments, mercury is present in soils, water and food chains from complex sources [9-16]. On one hand, these soils have accumulated mercury naturally through time [9]. Human activities such as deforestation and agricultural land use can mobilize mercury from soils and vegetation [14,15,17,18]. Also, the use of metallic mercury in the gold mining process can contribute to mercury contamination in these areas [10-13,16].

According to specific methylation rates, mercury compounds in certain aquatic environments can be transformed into methylmercury, the most toxic mercury compound. This organic form of mercury is easily assimilated and accumulated into the food chains, with biomagnification along the trophic levels [10]. This is the main pathway for human exposure via fish consumption.

The most deleterious and studied health effects of methylmercury are neurological dysfunctions [19-22], especially from *in utero *exposure [4,19,23-29]. Also, immunotoxic and citotoxic damages have been shown [30-33], as well as cardiovascular deleterious effects [5,34-37]. It was not easy to reach a consensus regarding safe values for methylmercury exposure [38]. Overall, the tendency through time has been to lower the recommended level as much as possible, in order to minimize the health risk [39,40]. Based on *in utero *neurological development, the Joint FAO/WHO Expert Committee on Food Additives suggested a Benchmark Dose Limit (BMDL) of 14 μg/g of mercury in maternal hair, recommending a daily mercury intake lower than 1.5 μg/kg of body weight [41]. The Environmental Protection Agency (EPA) stated the Reference Dose (RfD) at 0.1 μg/kg/day, expecting no more than 11 μg/g of mercury in maternal hair [42]. Most researchers have handled for Amazonian populations a BMDL of 10 μg/g of hair mercury [36,43].

Amazonian riverside populations, which are amongst the highest fish consumers in the world, are exposed to mercury because of their alimentary habits. This situation has been widely studied in Brazil and French Guyana since the late eighties, showing quite complete information about the determinants of mercury exposure in these riverside populations [38,44-51]. However, there is an evident lack of data in the rest of the Amazonian countries, where there have been a few isolated studies.

We aimed to systematize the available data and extract relevant information regarding hair mercury levels and observed health effects in Amazonian populations. We focused on the spatial distribution trends, in order to compare hair mercury levels by river basin and studied population.

## Methods

We used scientific databases and online libraries such as PubMed, ISI Web of Knowledge, ScienceDirect, SpringerLink, SciELO, Horizon, selecting articles with "Mercury, methylmercury, Amazon, hair, human exposure, fish consumption" as main keywords. We included the websites of international public institutions such as the Environmental Protection Agency (EPA), the Agency for Toxic Substances and Disease Registry (ATSDR), the World Health Organization (WHO) and the Food and Agriculture Organization (FAO).

The articles were included in this review if a measure of the hair mercury concentration was presented from the general population or a target group of an Amazonian region. We also considered similar basins in Brazil such as the Tocantins River and Guyana Plateau, because of their geographic and ecological interfaces with the Amazon regions.

Exclusion criteria were sample sizes less than 10 people, and the absence of a measure of central tendency (mean, geometric mean or median). Also, we excluded studies where the hair mercury concentration presented was an estimate calculated from the mercury concentration in fish tissue and the frequency and amount of fish consumption. In the cases where we found two or more articles corresponding to the same study and in the same population, we chose one to be represented on the maps.

We presented the results from all the selected articles in tables, showing author, study sites, sample sizes, hair mercury levels and fish consumption measures when available. The tables were organized by regions and using the same continuity given by the maps, starting with studies from Andean Amazonian countries (Table 1) and French Guyana (Table 2). Brazilian studies are presented in the following tables, divided according to the studied population or group (Tables 3, 4, 5). The studies conducted in the Tapajós River basins were presented in independent tables, also separated according to the studied population or group (Tables 6, 7, 8, 9). Finally, the studies from the Madeira River basin were displayed separately in the last table (Table 10).

**Table 1:** Hair mercury levels in Andean Amazonian Countries

Studied population	Study	N	Hg mean μg/g	Range	**> 10 μg/g**^a^	Fish
Country, River Basin			(median) [SD]	μg/g		
**General population**						
Barbieri et al. 2009	Cachuela Esperanza	150	3.76 (3.01) [2.52]	0.42-15.65	3%	10.5 meals/week
Bolivia, Beni River						
						
Monrroy et al. 2008	Upper Beni River	556	5.3 (4.0) [4.3]	0.08-34.	≈ 14.0%	
Bolivia, Beni River	Children	393	5.2 (3.9) [4.4]	0.08-34.1		
	Mothers	163	5.5 (4.4) [4.1]	0.15-20.0		
	Pregnant women	18	3.2 (3.3) [2.1]	0.2-7.8		
	Breastfeeding (BF)	57	6.2 (5.5) [4.1]	0.5-18.3		
	Non pregnant, non BF	93	5.4 (4.1) [4.1]	0.15-20.0		
						
						
Maurice-Bourgoin et al. 2000	Rurrenabaque	80				
Bolivia, Beni River	Esse-Ejjas indigenous	37	9.8	4.3-19.5		
						
						
Webb et al. 2004	Coca	45	1.9(1.5)	0.03-10.0		7.5 meals/month
Ecuador, Napo River	Añangu	27	8.7 (7.8)	2.2-20.5		17.2 meals/month
	Pañacocha	27	5.3 (5.0)	1.5-13.6		33.9 meals/month

						

**Children**						
Counter et al. 2005	Nambija gold-mining	80	2.8^b ^(2.0) [17.5]	1.0-135.0	<10%	
Ecuador, Nambija River	settlement					

^a^Percentage of the population with hair mercury levels higher than 10 μg/g. ^b^Geometric mean.

**Table 2:** Hair mercury levels in French Guyana

Studied population	River basin	Location	N	Hg mean μg/g (median) [SD]	Range μg/g	> 10 μg/g^a^	Fish consumption
**General ** **population**							

BASAG 2007	Maroni River	Sinnamary	285	1.8		5%	
	Oyapok River	Lower Maroni River	740	1.7-3.6		2.4%	
		Lower Oyapok	144	1.5-3.4		1 person	
		River	181	4.6-7.2		7.6-18.2%	
		Upper Oyapok River					

Cordier et al. 1998	Maroni River Oyapok River	11 health centers					
		Adults	255	3.4^b^	0.2-22.0	12.2%	
		Pregnant women	109	1.6^b^	0.2-13.0	4.6%	
		Children	136	2.5^b^	0.2-31.0	11.8	

Fréry et al. 2001	Maroni River	Cayodé, Twenké, Taluhen and Antécume-Pata	235	11.4 [4.2]	1.9-27.2	57.4%	20-317 g/day


**Mothers and their infants**							
Cordier et al. 2002	Maroni River Oyapok River	Upper Maroni River					
		Children	156	10.2^b^		79%	2 meals/day
		Mothers	90	12.7^b^			
		Oyapok River					
		Children	69	6.5^b^			
		Mothers	63	6.7^b^			
		Lower Maroni River					
		Children	153	1.4^b^			
		Mothers	77	2.8^b^			

^a^Percentage of the population with hair mercury levels higher than 10 μg/g. ^b^Geometric mean.

**Table 3:** Hair mercury levels in Brazilian River Basins, General Population

Study River Basin	Location	N	Hg mean μg/g (median) [SD]	Range μ g/g	**> 10 μg/g**^a^	Fish consumption
Akagi et al. 1995	Três Bocas	11	28.1	8.4-54.0		
Araguari River						

Castro et al. 1991	Surucucus, Paapiu and Mujacai areas	162	3.61	1.4-8.1		
Branco River						

Forsberg et al. 1995	Various sites between Marie and Paduari Rivers	154	75.5 [35.2]	5.8-171.2		
Negro River						

Kehrig et al. 1998	Balbina Reservoir	53	6.5 [5.4]	1.2-22.0		110 g
Negro River						

Leino & Lodenius 1995	Tucuruí area	125	35.0 (29.0)	0.9-240.0		11 meals/week
Tocantins River						

Pinheiro, Nakanishi et al. 2000	Belém	13	2.0			
Tocantins River						

Santos, Camara et al. 2002	Caxiuanã	214	8.6 [6.3]	0.6-46.0		12.3 meals/week
Amazon River						

Santos et al. 2003	Lower Mamoré: Pakaás Novos Indigenous Areas	910	8.4 (6.9) [6.4]	0.5-83.9		
Mamoré River						

Soares et al. 2002	Doutor Tanajura	13	(6.1)	1.4-11.7		
Mamoré River						

Vasconcellos et al. 1994	Indigenous Xingu Park	27	18.5 (18) [5.9]	6.9-34.0		
Xingu River	Billings Dam	28	0.9 (0.7) [0.7]	0.3-3.0		
	Controls	25	1.1 (1.0) [0.6]	0.3-2.5		

Vasconcellos et al. 2000	Xingu Park (13 groups)			1.2-57.3		
Xingu River	Highest values		21.8 (20.8) [6.1]			
	Lowest values		3.6 (2.6) [2.4]			

The Tapajós and Madeira Rivers basins are not displayed in this table (see Tables 5, 6, 7, 8, 9, 10). ^a^Percentage of the population with hair mercury levels higher than 10 μg/g.

**Table 4:** Mercury levels in Brazilian River Basins, Target Groups


**Studied population ** **River Basin**	**Location**	**N**	**Hg mean μg/g ** **(median) [SD]**	**Range ** **μg/g**	**> 10 μg/g^a^**	**Fish ** **consumption**

**Adults**						
Barbosa et al. 2001	Negro River shores	76	21.4 (17.8) [12.7]	1.7-59.0	79%	
Negro River	Men	17	26.2 [13.7]			
	Women	31	18.3 [11.1]			

Pinheiro et al. 2006	Panacauera	22	≈ 7.0			
Tocantins River	Pindobal Grande	43	≈ 3.0			

Silva et al. 2004	Tabatinga	98	(6.4)	1.2-17.0		
Amazon River, lakes						

Yokoo et al. 2003	Pantanal region	129	4.2 (3.7) [2.4]	0.6-13.6		
Cuiabá River						

						

**Children**						
Barbosa et al. 2001	Negro River shores	73	18.5 (16.4) [10.0]	0.5-45.9	79%	
Negro River						

Pinheiro et al. 2006	Pindobal Grande	88	≈ 3.0			
Tocantins River						

Pinheiro et al. 2007	Panacauera	36	2.3	0.4-9.5	0%	
Tocantins River						

Santos-Filho et al. 1993	Cubatão Municipality	217	0.8 [0.5]	0.2-3.0	0%	
Cubatão River						

Tavares et al. 2005	Barão de Melgaço	114	2.1 (1.8) [1.4]	0.4-7.6	0%	4.6 meals/week
Cuiabá River	Riverside communities	72	5.4 (4.7) [3.4]	0.6-17.1		7.8 meals/week

						

**Women**						
Pinheiro et al. 2008	Panacauera	20	3.3	1.3-6.0	0%	
Tocantins River						

						

**Mothers and ** **their infants**						
Barbosa et al. 1998	*Garimpo * Maria Bonita					
Fresco River (Xingu basin)	Mothers	28	8.1 (8.3) [3.2]	0.8-13.7		
	Infants	54	7.3 (6.6) [3.5]	2.0-20.4		

The Tapajós and Madeira Rivers basins are not displayed in this table (see Tables 5, 6, 7, 8, 9, 10). ^a^Percentage of the population with hair mercury levels higher than 10 μg/g.

**Table 5:** Hair mercury levels in Brazilian River Basins, Occupational Groups

Study	Location	N	Hg mean μg/g	Range	> 10 μg/g^a^	Fish consumption
River Basin			(median) [SD]			
Guimarães et al 1999	Pracuúba Lake	15	16.7			
Tartarugal Grande River (Fishermen)	Duas Bocas Lake	15	28.0		87%	14 meals/week (200 g per day)
	**Groups**					
Palheta & Taylor 1995	Garimpeiros	20		0.4-32.		
Gurupi River	Cachoeira Villagers	5		0.8-4.6		
	River dwellers	10		0.2-15		

The Tapajos and Madeira Rivers basins are not displayed in this table (see Tables 5, 6, 7, 8, 9, 10). ^a^Percentage of the population with hair mercury levels higher than 10 μg/g.

**Table 6:** Hair mercury levels in the Tapajós River Basin, General Population

Study	Location	N	Hg mean μg/g (median) [SD]	Range μg/g	> 10 μg/g^a^	Fish consumption
Akagi et al. 1995	Rainha	11	15.8	2.4-31.0		
	Brasília Legal	56	22.6	3.5-151.0		
	Ponta de Pedra	10	10.2	6.2-12.6		
	Jacareacanga	48	16.6	1.5-46.0		
						
Barbosa et al. 1997	Apiacás Reservation	55	34.2 (42.8)	?-128	93%	≈ 6 times/week

Crompton et al. 2002	Jacareacanga	205	8.6	0.3-83.2		

Dorea et al. 2005	Kaburuá	89	2.5 [1.4]			22 g/day
	Cururu Mission	138	3.7 [1.6]			32 g/day
	Terra Preta	22	6.0 [2.9]			52 g/day
	Kayabi	47	12.8 [7.0]			110 g/day

Malm et al. 1995	Jacareacanga	10	25.0	5.7-52.0		
	Brasília Legal	13-29	26.0	4.7-151.0		
	Ponta de Pedra	4-26	12.0			
	Santarem	11	2.7			

Pinheiro, Guimarães et al. 2000	Rainha	29	17.2			
	Barreiras	111	18.9			
	São Luís do Tapajós	30	25.3			
	Paranα-Mirim	21	9.2			

Pinheiro, Nakanishi et al. 2000	Rainha	29	17.6			
	Barreiras	78	19.1			

Santos et al. 2000	Brasília Legal	220	11.8 [8.0]	0.5-50.0		10 meals/week
	São Luís do Tapajós	327	19.9 [12.0]	0.1-94.5		13 meals/week
	Santana do Ituquí	321	4.3 [1.9]	0.4-11.6		13 meals/week

Santos, Camara et al 2002	Santana do Ituquí	321	4.3 [2.2]	0.4-12.0		12.7 meals/week
	Aldeia do Lago Grande	316	4.0 [2.1]	0.4-12.0		12.0 meals/week
	Vila do Tabatinga	499	5.4 [3.1]	0.4-17.0		10.5 meals/week

Santos, de Jesus et al. 2002	Sai Cinza	324	16.0 [18.9]	4,5-90,4		

Silva et al. 2004	Jacareacanga	140	(8.0)	0.3-58.5		
	Rio-Rato	98		0.01-81.4		

^a ^Percentage of the population with hair mercury levels higher than 10 μg/g.

**Table 7:** Hair mercury levels in the Tapajós River Basin, Adults

Study	Location	N	Hg mean μg/g (median) [SD]	Range μg/g	> 10 μg/g^a^	Fish consumption
Amorim et al. 2000	Brasília Legal	98	(13.5)	0.6-71.8	>50%	
	Women		(10.8)			
	Men		(17.1)			
						
Dolbec et al. 2000	Cametá	68	10.8 (9.0) [6.1]			61.8% of total meals
	Women	41	9.9 (8.0) [5.6]		>25%	
	Men	27	12.2 (10.8) [6.8]		>50%	
						
Fillion et al. 2006	São Luís do Tapajós, Nova Canaã, Santo Antônio, Mussum, Vista Alegre and Açaituba	251	17.8	0.2-77.2	69.7%	6.8 meals/week
						
Lebel et al. 1997	Brasília Legal	96	(12.9)		>50%	
	Women		(11.2)			44.7% of total meals
	Men		(15.7)			43.9% of total meals
						
Lebel et al. 1998	Brasília Legal					
	Men	34	14.3 [9.4]			
	Women	46	12.6 [7.0]			
						
Passos et al. 2007	SLTapajós, Nova Canaã, Santo Antônio, Ipaupixuna, Novo Paraíso, Teça, Timbu, Açaituba, Campo Alegre, Samauma, Mussum, Vista Alegre and Santa Cruz	457	16.8 (15.7) [10.3]	0.2-58.3	>50%	6.6 meals/week
						
Pinheiro et al. 2006	São Luís do Tapajós	32	≈ 15.0			
	Barreiras	37	≈ 15.5			
						
Silva et al. 2004	Vila do Tabatinga	98	(6.4)	1.2-17.0		

^a^Percentage of the population with hair mercury levels higher than 10 μg/g.

**Table 8:** Hair mercury levels in the Tapajós River Basin Women and Children

Studied population	Location	N	Hg mean μg/g (median) [SD]	Range μg/g	> 10 μg/g^a^	Fish consumption
**Mothers and ** **their Children**						
Grandjean et al. 1999	Mothers	114	11.6 (14.0)			
	Children		11.0 (12.8)	0.5-83.5		2 meals/day
	Brasilia Legal	76	11.9	0.7-35.8	76%	
	São Luís do Tapajós	71	25.4	0.6-83.5	91%	
	Sai-Cinza	87	17.7	7.3-63.8	92%	
	Santana do Ituquí	105	3.8	0.5-12.4	2%	

						

**Children**						
Barbosa et al. 1997	Apiacás Reservation	28	29		86%	
						
Dorea, Barbosa et al. 2005	Kaburuá	77	2.9 [2.1]			
	Cururu Mission	86	4.8 [2.1]			
	Kayabi	40	16.6 [11.4]			
Pinheiro et al. 2007	São Luís do Tapajós	48	10.9	1.3-53.8	52%	
	Barreiras	84	6.1	1.4-23.6	21%	

						

**Women**						
Barbosa et al. 1997	Apiacás Reservation	13	41.2		100%	
						
Dolbec et al. 2001	Cametá	98	(12.5)	2.9-27.0	>50%	
						
Hacon et al. 2000	Alta Floresta	75	1.12 [1.2]	0,05-8,2	0%	8-20 g/day
						
Passos et al. 2003	Brasília Legal	26	10.0 (9.1)	4.0-20.0	≈ 50%	8 meals/week
						
Pinheiro et al. 2007	São Luís do Tapajós					5-14 meals/week
	Rainha, and Barreiras					
	Pregnant	19	8.2	1.5-19.4	37%	
	Non-pregnant	21	9.4	5.2-21.0	28%	
						
Pinheiro et al. 2005	São Luís do Tapajós	28	13.7	3.2-30.04	36%	
	Barreiras	39	12.1	3.04-33.4	38%	

^a^Percentage of the population with hair mercury levels higher than 10 μg/g.

**Table 9:** Hair mercury levels in the Tapajós River Basin, Occupational Groups

Study	Locations and groups	N	Hg mean μg/g (median) [SD]	Range μg/g	> 10 μg/g^a^	Fish consumption
Harada et al. 2001	Fishermen and families					
	Barreiras	76	16.4 [10.6]	1.8-53.8	75%	
	Rainha	12	14.1 [9.3]	3.1-34.5	67%	
	São Luís do Tapajós	44	20.8 [10.6]	5.1-42.2	86%	
						
						
Lebel et al. 1997	Brasília Legal					
	Fishermen	14	(27.3)		> 50%	68.8% of total meals
Lebel et al. 1998	Brasília Legal					
	Fishermen	11	23.9 [9.3]			
						
						
Santos, de Jesus et al. 2002	Sai Cinza	324	16.0 [18.9]	4,5-90,4		
	Agriculture	127	17.3	6.8-90.4		
	Gold mining	6	13.8	10.7-18.5		
	Both of the above	25	13.8	6.9-25.7		
	Others	11	12.3	6.6-20.6		
	Children up to 6 y/o	93	16.8	4.5-66.6		
	Students	38	15.1	9.0-38.7		
	Without information	24	17.0	9.6-30.8		

^a^Percentage of the population with hair mercury levels higher than 10 μg/g.

**Table 10:** Hair mercury levels in the Madeira River Basin

Studied population	Location	N	Hg mean μg/g (median) [SD]	Range μg/g	> 10 μg/g^a^	Fish consumption
**General population**						
Bastos et al. 2006	Various populations along the Madeira River	713	15.2 (12.5) [9.6]	0.36-150.0	>50%	7 meals/week
						
Boischio & Barbosa 1993	Near Porto Velho	311	≈ (10)	?-303.1	51%	200 g/day^b^

						

**Mothers and their infants**						
Barbosa & Dorea 1998	Near Porto Velho					
	Mothers	98	14.1 (12.8) [10.7]	2.6-94.7	>50%	
	Infants	71	10.8 (7.8) [8.5]	0.8-44.4		
						
Boschio & Cernichiari 1998	Near Porto Velho					
	Mothers	12		4.0-41.0		
	Infants	12		8.2-50.4		
						
Boischio & Henshel 2000	Near Porto Velho					
	Mothers	90	12.6 [6.5]	15.0-45.0		
	Infants	89	10.2 [7.2]	1.0-34.2		
						
Marques et al. 2007	Porto Velho city					
	Mothers	82	(5.4)	0.4-62.4		1 meal/week
	Infants	82	(1.8)	0.02-32.9		

^a^Percentage of the population with hair mercury levels higher than 10 μg/g. ^b^From the Letter to the Editor Boischio Environ Research 2000; 82:91-92

In the cases when an article studied populations from different river basins, we presented them separately, in the corresponding basin tables.

For the design of the maps, we located each Amazonian study site using their longitude and latitude data when available in the article. Otherwise, we searched longitude and latitude data in geographic databases and using Google Earth, which allowed us to find and successfully locate the majority of the study sites. When it was not possible to identify the coordinates, we used the maps and/or site descriptions presented by the authors. We marked and named each study site on the map displaying a representation of the hair mercury levels found in this population in a six colour scale. Each individual square on the map represents a hair mercury measure and the target group used for the study, as well as the reference number. We prepared separated maps for the Madeira River, the Tapajós River and French Guiana. We also designed a map illustrating the studies that assessed health outcomes in relation to hair mercury levels in the studied populations, using a three colour scale for the different results.

## Results and Discussion

**Figure 1:**
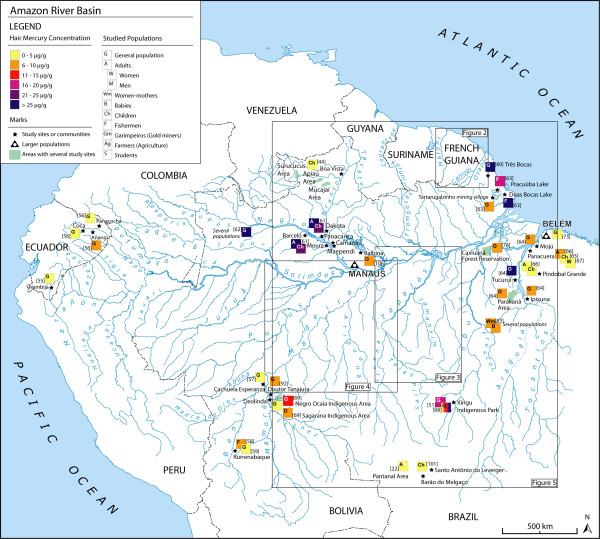
**Hair mercury levels in the Amazon River basin**.

**Figure 2:**
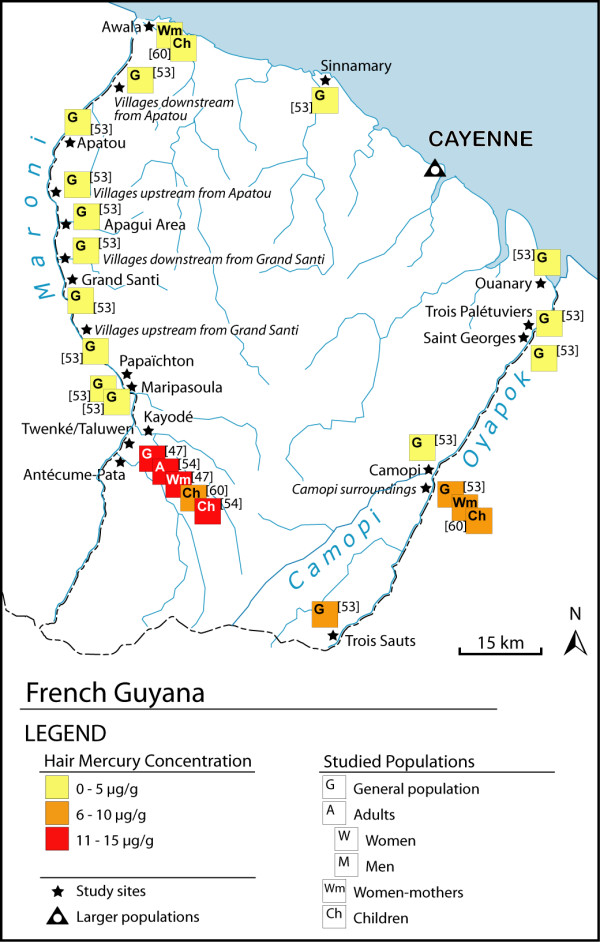
**Hair mercury levels in French Guyana**.

**Figure 3:**
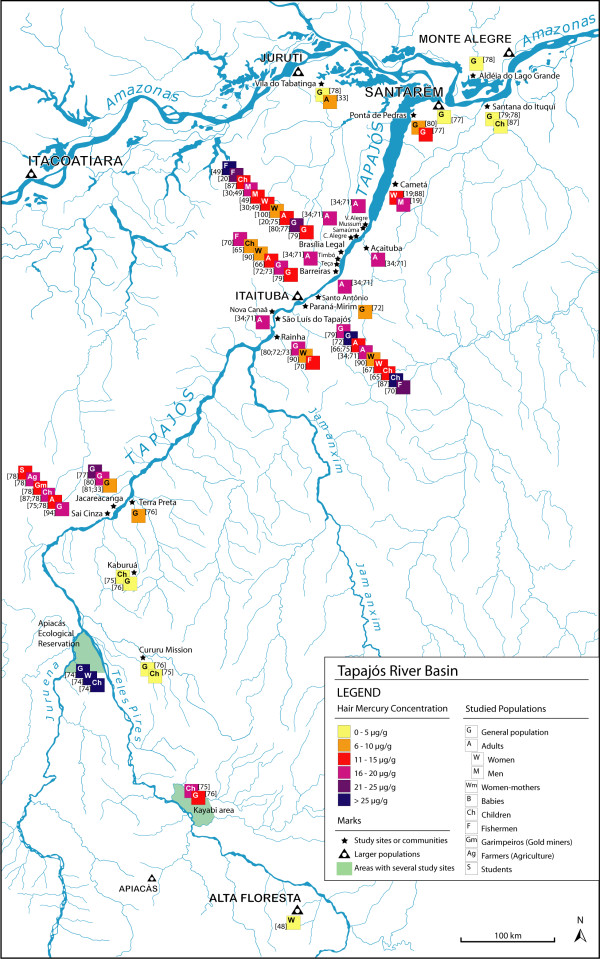
**Hair mercury levels in the Tapajós River basin, Brazil**.

**Figure 4:**
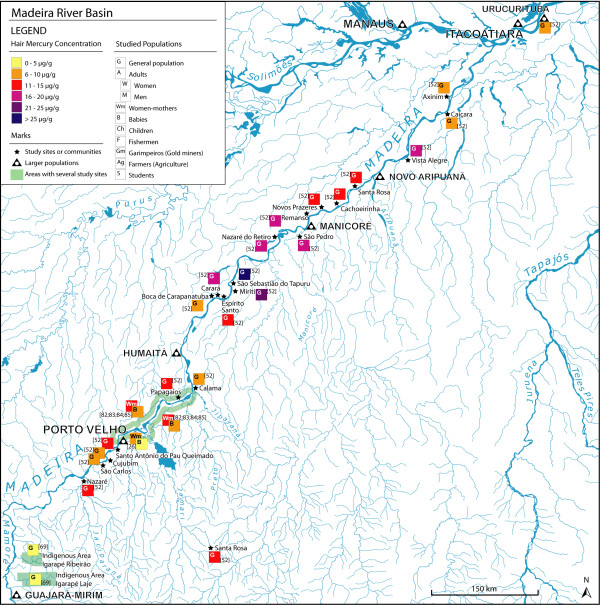
**Hair mercury levels in the Madeira River basin, Brazil**.

We found 58 articles meeting our criteria for the elaboration of the maps. The majority of the studies were carried out in Brazil (86%), while there are only 3 studies in French Guyana, 3 in Bolivia and 2 in Ecuador (Figures 1, 2). In Brazil, 30 studies are focused on the Tapajós River basin (Figure 3), 10 on the Madeira River basin (Figure 4) and the remaining 20% of these Brazilian studies are from other basins, such as the Negro River, the Tocantins River or the Xingu River (Figure 1).

The approaches and strategies are quite variable between studies. Several studies have used a large sample size, selected randomly from the general population (Tables 1, 2, 3, 6, 10), and usually comparing two or three sites from the same basin. Some of them used smaller sample sizes, accepting the variability induced by the sampling fluctuation in order to extend to several populations geographically close to each other. In the Madeira River (Figure 4; Table 10), the study by Bastos et al. (2006) covered from the upper basin (near the Bolivian border) to a point downstream on the Amazon River [52]. Even if some of the populations had sample sizes of less than 10 people, the locations evaluated were 44, distributed along the largest part of the Madeira basin (Figure 4).

In French Guiana the studies have used both approaches, covering almost the totality of the Maroni and Oyapok River basins, with large sample sizes from the general population (Figure 2; Table 2) [47,53,54].

### Hair mercury levels in the Amazon

In the Andean Amazonian regions (Figure 1; Table 1), mercury levels were found below 10 μg/g in Ecuador [55,56] and Bolivia [57-59].

In French Guiana (Figure 2; Table 2), most study sites showed hair mercury levels below 10 μg/g in the Oyapok River and the lower Maroni River basins [53,60]. The only populations with mercury levels above 10 μg/g were located in an Amerindian reservation in the upper Maroni River basin [47,53,54].

In Brazilian Amazonia (Figures 1, 3, 4; Tables 3, 4, 5) hair mercury levels are very variable between basins and study sites. Without considering the studies from the Tapajós and Madeira Rivers, which will be explained in detail below, the highest mercury levels can be found in the Negro River basin [61,62] and at the lakes from the Amapá State (Figure 1; Tables 3, 4, 5) [63], with means above 20 μg/g and even higher than 25 μg/g. These high values can also be found in two other sites from the Xingu and Tocantins Rivers, both from Amerindian reservations (Figure 1) [51,64-68]. These levels can be considered of a high risk for the populations and would merit further investigations about the health impact of this exposure. The rest of these study sites show hair mercury levels below 10 μg/g, with the exception of an Amerindian reservation on the Mamoré River (13.1 μg/g) [69].

#### Tapajós River basin

In the Tapajós River basin (Figure 3; Tables 6, 7, 8, 9) there is a wide difference in mercury levels between the various populations. Most locations present hair mercury levels above 10 μg/g, especially in some populations such as Rainha, Barreiras, Brasília Legal or São Luís do Tapajós [34,70-73], where exposure levels seem to be alarming, even reaching a mean over 30 μg/g in the Apiacás Reservation study [74]. In very few sites the hair mercury levels remained below 5 μg/g [75,76]. The populations living in urban or suburban areas (Santarém and Santana do Ituquí) were used as control populations by the authors and showed lower exposure levels attributed to their food diversification [33,77-79]. In the same way, some authors used study sites from different river basins to compare their exposure levels, such as Panacuera or the city of Belém (Figure 1) [65,73].

Unexpectedly, studies carried out in the same site and target group obtained quite different results. For example, in Jacareacanga the studies on the general population published in 1995 [77,80] showed much higher levels (means of 16.6 and 25.0 μg/g) than those carried out in the following decade (mean = 8.6 μg/g and median = 8.0 μg/g) [33,81]. The sample sizes and study designs were very different. In 1995, Jacareacanga was a village with approximately 3000 inhabitants, and the two studies published that year took samples of 10 and 48 people. In 2002, the population of Jacareacanga was around 2000 inhabitants, and the studies published in 2002 and 2004 took samples of 140 and 205 people. Other than that, there is not enough information to explain the observed difference in hair mercury levels. There is no evidence of a change in the fish consumption frequency, deforestation, colonization, agricultural land use or gold mining activities.

#### Madeira River basin

In the Madeira River basin (Figure 4; Table 10) most study sites showed hair mercury levels between 11 μg/g and 15 μg/g [52,82-85]. A couple of sites (very close to each other) presented hair mercury levels above 20 μg/g [52]. A few sites showed levels between 16 μg/g and 20 μg/g and the others presented hair mercury levels under 10 μg/g [52,82,83,85]. This shows a mercury exposure distribution that appears to be less heterogenic than the Tapajós River basin. The higher mercury levels seem to be found in small isolated villages on the middle of the basin, downstream from Humaitá, near Manicoré (Figure 4).

### Mercury levels and fish consumption

In the Brazilian Amazon, many studies include especially fish consumption in the population, even though not all of them show these results (Tables 1, 2, 3, 4, 5, 6, 7, 8, 9, 10). Fish consumption is not measured in a uniform manner in all the studies. Each research team chose their indicators according to their objectives, so this measure can be found in grams per day, percentage of meals composed by fish, meals per week or per day and/or times per week. No matter which indicator for fish consumption the authors chose, there was always a positive relation between fish consumption and hair mercury levels [19,46,47,49,64,75,76]. Most of these study sites are usually small and traditional riverside villages, some of them Amerindian reservations, without the proper roads connecting them to larger villages and cities. It is possible to hypothesize that this situation makes those populations more dependent on fish as source of protein intake.

In spite of those considerations, some populations with high fish consumption showed hair mercury levels below 10 μg/g, even if there was a significant relation with fish consumption frequency [55-57,59,78,79]. In those particular six studies, it is important to consider the different geographical and socio-economic situation those populations are in: three of those studies were conducted in the Andean piedmont Amazonian regions, the two Ecuadorian studies [55,56] and one of the Bolivian studies [57]. Also, those populations are not solely dependent on fish because they are also farmers or hunters, even if fish remains the most important source of nutrients. The Bolivian population of Cachuela Esperanza, located near the Brazilian border, consumes large amounts of fish (10 fish meals per week), but only during the dry season, preferring game meat during the rainy season, which lasts from October to April [57].

The other two cases with intense fish consumption and low hair mercury levels are the studies conducted in Aldeia do Lago Grande, Santana do Ituquí and Vila to Tabatinga. These communities are located on the Amazon River shore, near the confluence with the Tapajós River. They are small but not isolated because they are connected by roads to the cities of Monte Alegre, Santarém and Juruti. The low hair mercury levels cannot be explained by food diversification, because these populations consume 10 to 13 fish meals per week. However, the fish they consume is collected from local lakes and minor rivers, and the mercury concentrations in fish tissue was found to be much lower than in other exposed populations [52,78,79].

On the Madeira River basin, where most hair mercury levels remained under 10 μg/g, the fish consumption in the majority of the populations was around seven meals per week (Table 10). On the contrary, on the Tapajós River basin the fish consumption in the majority of the populations was higher than 10 meals per week (Table 6).

Some studies measured mercury concentrations in fish tissue, finding positive relations between fish mercury concentrations, fish consumption and human hair mercury levels [13,75,77,80,86].

### Studied populations

Due to the importance of mercury exposure and its health effects in children, as well as *in utero *exposure, many studies have chosen fertile age women and children as target groups (Tables 1, 2, 4, 8, 10). These studies are generally consistent with studies on the general population, with comparable hair mercury levels. When studied together as mother-infant pairs, there was always a strong relation between maternal hair mercury and the exposure in their infants; the mothers always had higher mercury levels than their infants [26,60,82,83,85,87].

The majority of the studies did not find any significant relationship between age and hair mercury levels [13,30,33,44,48,61,66,77,78,85,88-92]. Some studies found that hair mercury levels increase with age [74,78,79,81,84]. Nevertheless, a couple of studies found the opposite results, showing higher hair mercury levels in younger people, children and infants [58,69].

Several studies found that men have higher hair mercury levels than women [19,30,34,61,65,69,71,74,84,87]. In two articles from the Tapajós River basin, only the fishermen had hair mercury levels significantly higher than the women, while the other men from the same village presented hair mercury levels similar to those of the women (Tables 7, 8). Those differences corresponded to their fish consumption, which was also significantly higher for the fishermen [20,49]. Only one study found women to have higher mercury levels than men [69].

No study revealed physiological basis that could lead to hypothesize a difference between female and male mercury metabolism. In fact, most studies (78%) did not find any significant relation between mercury exposure and gender.

A few studies focused their attention on fishermen, because of their obvious access to fish as main nutrient [49,63,70]. Their hair mercury means ranged from 16 to more than 25 μg/g, regardless of the river basin. For instance, the studies conducted by Lebel et al. (1997, 1998) compared fishermen and other adults from the Brasilia Legal population. Both men and women had mercury levels lower than 15 μg/g, while fishermen presented levels of 27.3 μg/g and 23.9 μg/g (Figure 3; Tables 7, 8) [20,49].

The particular case of gold miners (called *garimpeiros *in Brazil and Bolivia) has been documented in a couple of studies. It is important to remark that occupational mercury exposure is very different from the environmental exposure. Artisanal *garimpeiros *are occupationally exposed to metallic mercury vapours, which are rapidly transformed in the human body into inorganic mercury, best measured in urine or plasma, while total hair mercury corresponds mainly to methylmercury exposure in high fish eating populations [36,93]. Generally, the authors found that *garimpeiros *had lower hair mercury levels than the *ribeirinhos *[58,94], concluding that because of their better economical situation, they were able to diversify their food, consuming less fish than the rest of the population[13]. However, in a few studies the *garimpeiros *or gold miners had higher total mercury levels in hair than the general population, without apparent relation with fish consumption frequency [45,57,95,96]. Consistently, in those three studies the hair mercury means in the general population were lower than those found in most central Amazonian studies. In both the Bolivian study and the Colombian study, hair mercury means remained below 6 μg/g, even in the most exposed groups [57,95]. It would be possible to assume that metallic mercury exposure can acquire significance in hair mercury levels in some specific situations of low methylmercury exposure. However, considering the differences between those studies and regions, and without hair mercury speciation, it is not possible to reach valid conclusions.

### Multidisciplinary studies

Five studies carried out a multidisciplinary approach, measuring as well mercury in air, water, sediments, fish and human samples [13,52,58,63,96]. In those studies, human exposure seemed to be considered as a part of the totality of the environmental contamination. Therefore, those hair mercury levels were useful as a reference value for those specific study sites, and need to be interpreted in the whole context rather than as a Public Health assessment. These studies showed in a precise manner the interactions within various environmental components, showing a direct relation between mercury concentrations at different levels of the ecosystem and the food chains, including the human being as the last receptor of this pollution.

An example of an ecosystem approach is the CARUSO project [14,15,17-20,30,34,49,71,88,97-100]. This project started in 1994, and consists on a series of studies conducted by research teams of different disciplines, attempting to assess the various aspects of mercury pollution and human exposure in the Tapajós River basin.

### Hair mercury levels and health impact

**Figure 5:**
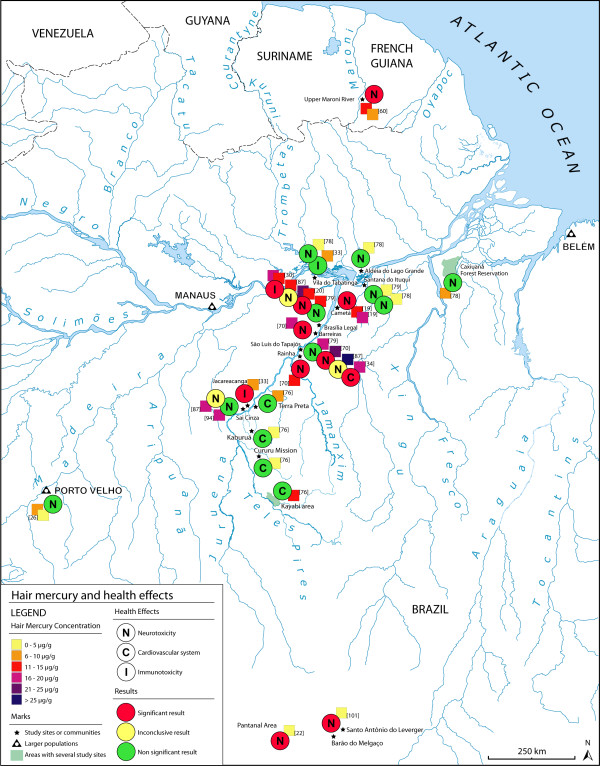
**Hair mercury levels and health impact in the Amazon River basin**.

There were 15 studies conducted in Brazil and one in French Guyana (Figure 5). Most of them (73%) focused on neurological effects.

In the Amazonian context it is not easy to identify a clear relation between hair mercury levels and patent neurological abnormalities. Observing the spatial distribution of the studies, we can see that in populations with low hair mercury levels, such as Santana do Ituquí, Vila do Tabatinga or the city of Porto Velho, it was not possible to confirm a relation between mercury levels and neurological performance [26,78,79,101]. Also, in the populations with higher mercury exposure, the authors found an impact on the nervous system, especially motricity [19,20,60].

However, in São Luís do Tapajós or Brasília Legal the information is difficult to interpret. There were two studies assessing neurotoxicity in the general population and one in adults. While one of those studies documented cases of mild Minamata disease [70], the other did not find any relation between hair mercury levels and neurological outcomes [79]. On the contrary, the study conducted in adults found a dose-dependent relation between hair mercury levels and motor abnormalities [20]. There was also a study that found an association between exposure and psychomotor performance in children, but the authors concluded that the outcomes observed were also influenced by socioeconomic factors, such as maternal educational level or nutritional status [87].

Two studies have evaluated the impact of mercury exposure on the immune system *in vitro*. The authors found positive relations between mercury levels and the presentation of auto-antibodies [33] and cytogenetic abnormalities in peripheral lymphocytes [30], especially in the most exposed Amazonian populations.

There are a couple of studies about mercury exposure and blood pressure on the Tapajós River basin. One of them supports a negative impact of mercury exposure on the cardiovascular system [34], while the other did not find a significant relation between mercury levels and blood pressure [76]. This second study was conducted in four Amerindian populations located on rivers tributaries to the Tapajós, with low hair mercury levels. The authors found that the community with higher hair mercury levels (12.8 μg/g) seemed to be protected against age-related increase of blood pressure, because of their intense fish consumption [76].

Two studies were focused on children's growth. One of these studies was carried out in an Amerindian community from the Tapajós River [75]. No significant correlation was found between growth or nutritional status and hair mercury levels. On the contrary, a study in the Bolivian Amazonia found a positive relation between hair mercury levels and a better nutritional status in children ranging from 5 to 10 years of age [102]. Considering the characteristics of that population, the authors hypothesized that the relation observed was caused by the nutritional contents in fish, mostly important at that age group. Furthermore, the same study found the opposite result in the mothers, with worse nutritional indices in the women with higher mercury levels. In fact, while some authors worry about the health impact of mercury exposure in high fish eating populations [38,99,103], others consider that the nutritional value of fish probably compensate an exposure to low mercury levels [46,75,102,104,105]. In absence of large cohort studies, as the ones developed in the Faeroe Islands and Seychelles, the issue of health impact in the Amazonian context still remains open for debate.

### Other interactions

Interest has recently been focused on the relation between mercury exposure and antioxidant defences, suggesting that long term mercury exposure induced a depletion in the antioxidant enzymatic activity [67].

Interactions between fish and fruit consumption were studied in a population from the Tapajós River, finding an inverse relationship between fruit consumption and Hg levels [71,100]. The authors recommended further investigation to establish new nutritional strategies aiming to limit mercury exposure while maintaining fish consumption.

Recently, some studies also emphasized the interactions between mercury and selenium from dietary sources, especially fish consumption [68,90,92,98]. The authors generally found high concentrations of selenium in the populations exposed to methylmercury, with molar ratios consistently close to 1. Based on their observations, the authors suggest the need of further research regarding the protective role of selenium against mercury toxicity.

### Variability in exposure assessment

As seen on the maps and tables, there is a considerable spatial and temporal variability in the measure of mercury exposure. Fish consumption frequency alone does not explain the differences in hair mercury levels in some study sites. There are several factors involved: the characteristics of the soils, methylation rates in different aquatic environments, the food chain structures, specific human habits and diets, social and economic characteristics, mercury interactions with other elements, and also strong local variations without a clear explanation. Moreover, many of these small populations are subjects to intense changes through time. Human migration, land use and deforestation are usually associated with erosion peaks, which could temporarily mobilize mercury from the soils [14,15,17,18].

It is also possible to attribute some of the variations observed to the use of hair as biomarker. It has been stated that methylmercury corresponds to more than 90% of total hair mercury [19,93,106]. Therefore, total mercury in hair has been widely accepted as a well-validated methylmercury exposure marker, reflecting non-occupational exposure by seafood or fish consumption. Nevertheless, a study by Barbosa et al. (2001) in a Negro River basin population found important variations in the percentage of methylmercury in total hair mercury, ranging from 34% to 100% [61]. This variation did not seem to be influenced by age, gender, body mass index, frequency of fish consumption or total hair mercury levels. Besides, it is worth mentioning that the use of this biomarker for toxicological purposes has been criticized for its lack of reproducibility [107,108].

That sum of factors makes it difficult to assess a certain situation with exactitude in a determined population. Also, it is important to consider the variability induced by the different sampling, target populations and methodologies used by the researchers according to their objectives. Therefore, we assume that not every hair mercury value represented on the maps and tables can be completely or accurately comparable to each other for epidemiological purposes.

## Conclusions

Considering the current recommendations from the environmental agencies, it is patent that almost all the Amazonian riverside populations are exposed to mercury contamination through alimentary habits. There is no evident spatial trend, even if the highest hair mercury levels were found in the central Amazonian regions, especially the Tapajós River basin. Small and isolated communities with traditional lifestyles seem to be the most exposed to mercury, regardless of the river basin. This situation is very complex for these populations, given that many of them depend on fish for economic support as well as almost unique source of dietary protein.

The available information about the health impact of this situation is not conclusive. Therefore, it becomes difficult to assess accurately the Public Health implication of mercury exposure in this particular context.

Besides, there are numerous Amazonian regions with lacking data and also with discordant results. Thus, a harmonized assessment of mercury human exposure based on a standardized approach would be valuable in order to spatially identify the most contaminated populations. This assessment would also allow prospective follow-ups of exposed populations, especially considering that these small communities are in constant evolution and subject of global changes.

## Competing interests

The authors declare that they have no competing interests.

## Authors' contributions

Both authors conceived the review, drafted the manuscript and designed the maps.
